# Data supporting the anticancer activity of posterior salivary gland (PSG) toxin from the cuttlefish *Sepia pharaonis* Ehrenberg (1831)

**DOI:** 10.1016/j.dib.2017.05.010

**Published:** 2017-05-05

**Authors:** Ramachandran Karthik, Venkatesan Manigandan, Kumar Ebenezar, Raghavan Vijayashree, Ramachandran Saravanan

**Affiliations:** a Department of Medical Biotechnology, Chettinad Academy of Research and Education, Kelambakkam 603103, Tamil Nadu, India; b Faculty of Allied Health Sciences, Chettinad Academy of Research and Education, Kelambakkam 603103, Tamil Nadu, India; c Department of Pathology, Chettinad Hospital and Research Institute, Kelambakkam 603103, Tamil Nadu, India

**Keywords:** PSG toxin, Anticancer, Anti-proliferative, Breast cancer, MTT assay, LDH leakage assay

## Abstract

The data presented illustrated the *in vitro* anti-proliferative effect of the PSG toxin from the cuttlefish, *Sepia pharaonis*. The cytostatic potentials of the PSG toxin were determined by the lymphocyte migration inhibition assay. The PSG toxin (50 μg/ml) exhibited commendable inhibition of the migration of lymphocytes across the agarose gel matrix under the presence of lipopolysaccharide mitogen, with a mean migration index of 0.625. The cytotoxicity of the PSG toxin against selected cancer cell lines was determined using the MTT assay. The PSG toxin exhibited dose-dependent cytotoxicity against the MCF-7 breast cancer cells followed by KB (oral), HeLa (cervical) and A549 (lung) cancer cell lines. The PSG toxin also exhibited proportional release of LDH leakage by mitochondrial damage with an IC_50_ of 13.85 μM against MCF-7 breast cancer cells. The *in vitro* anticancer activity of the PSG toxin against the selected cell lines was evaluated by Karthik et al. (2017) [1].

**Specifications Table**

**Table:** 

Subject area	Biology
More specific subject area	Breast cancer, *in vitro*, Anti-proliferative activity, PSG toxin
Type of data	Figure
How data was acquired	Nikon Eclipse Ti-U Inverted Microscope, USA; Bio-Rad PR4100, USA Microtiter plate reader.
Data format	Analyzed
Experimental factors	Peripheral blood mononuclear cells, MCF-7, KB, HeLa and A549 cancer cell lines were treated with PSG toxin from *S. pharaonis*.
Experimental features	**Lymphocyte migration inhibition assay**: Inhibition of LPS induced leucocyte migration by PSG toxin, observed in a Nikon Eclipse Ti-U Inverted Microscope, USA**MTT assay**: Reduction of MTT by mitochondrial succinate dehydrogenase in viable cancer cells treated with PSG toxin to a purple formazan product deteted in microplate reader**LDH release assay**: Reduction of the substrate lactate by LDH released into medium of cancer cells treated with PSG toxin, detected in microplate reader
Data source location	Faculty of Allied Health Sciences, Chettinad Academy of Research and Education, Chettinad Health City, Kelambakkam, Chennai, Tamil Nadu, India. 12.7948°N, 80.2160°E
Data accessibility	All data are provided with this article

**Value of the data**

• The provided data demonstrates the cytostatic potentials of the PSG toxin from *S. pharaonis* against peripheral blood mononuclear leucocytes.
• The data provided illustrates the commendable anti-proliferative action of the PSG toxin from *S. pharaonis* against the selected cancer cell lines.
• The data might be valuable to researchers interested in anti-proliferative action of toxins from marine mollusks.
• The data might also be of value to researchers investigating the anti-proliferative and anticancer activity of marine bioactive compounds.

## 1 Data

**Fig. 1:**
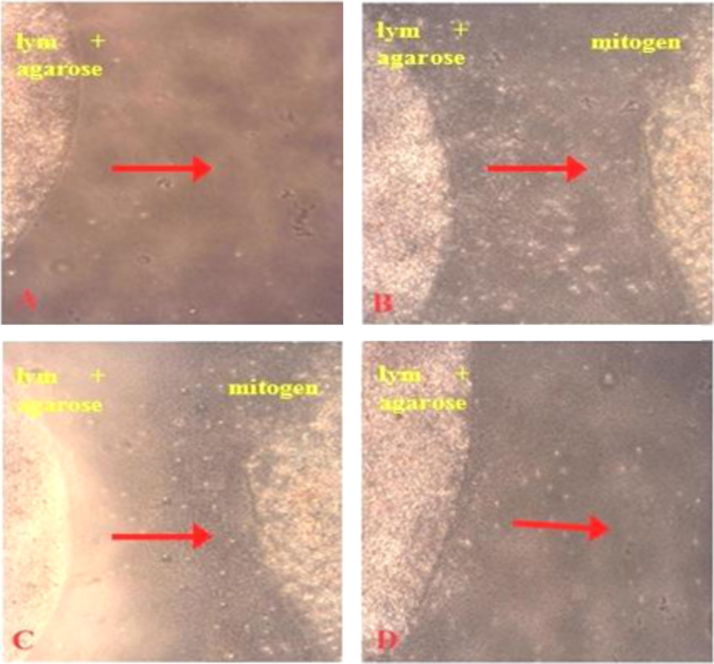
Cytostatic activity of PSG toxin by inhibition of lymphocyte migration. Migration inhibition of PSG toxin against leucocytes. (A) Control leucocyte-agarose droplet (B) Migration of leucocytes from agarose droplet towards mitogen (). (C) Migration inhibition in presence of PSG toxin (5 μg/ml) (D) Migration inhibition in presence of PSG toxin (50 μg/ml).

The migration of the lymphocytes was inhibited by the PSG toxin (5, 25 and 50 µg/ml) with a mean diameter of 1.65±0.55 mm and 1.42±0.64 mm respectively. The lymphocytes exhibited pronounced migration under the influence of LPS with a mean diameter of 3.45±0.83 mm (Fig. 1).

**Fig. 2:**
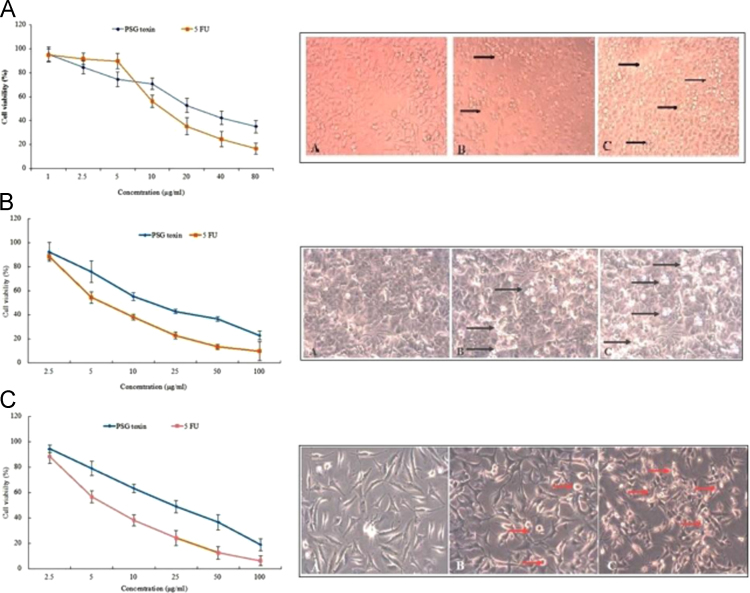
MTT cell viability curve of PSG toxin against (A) KB oral cancer cells (B) HeLa cervical cancer cells (C) A549 lung cancer cells. Microscopic images of (A) control, (B) PSG toxin treated and (C) paclitaxel treated cells indicating aggregation of apoptotic cells.

The cell viability curve of the PSG toxin against the KB oral cancer cells with an IC_50_ concentration of 32.5 µM. The purified PSG toxin also exhibited significant inhibition against the proliferation of HeLa cervical cancer cells and A549 lung cancer cells at an IC_50_ value of 16.5 µg/ml (23.2 µM) and 22.45 µg/ml (31.65 µM) (Fig. 2). The inlet figure shows the cell viability in (A) control, (B) PSG toxin (100 μg/ml) and (C) Paclitaxel treated cells showing the zones of apoptosis.

**Fig. 3:**
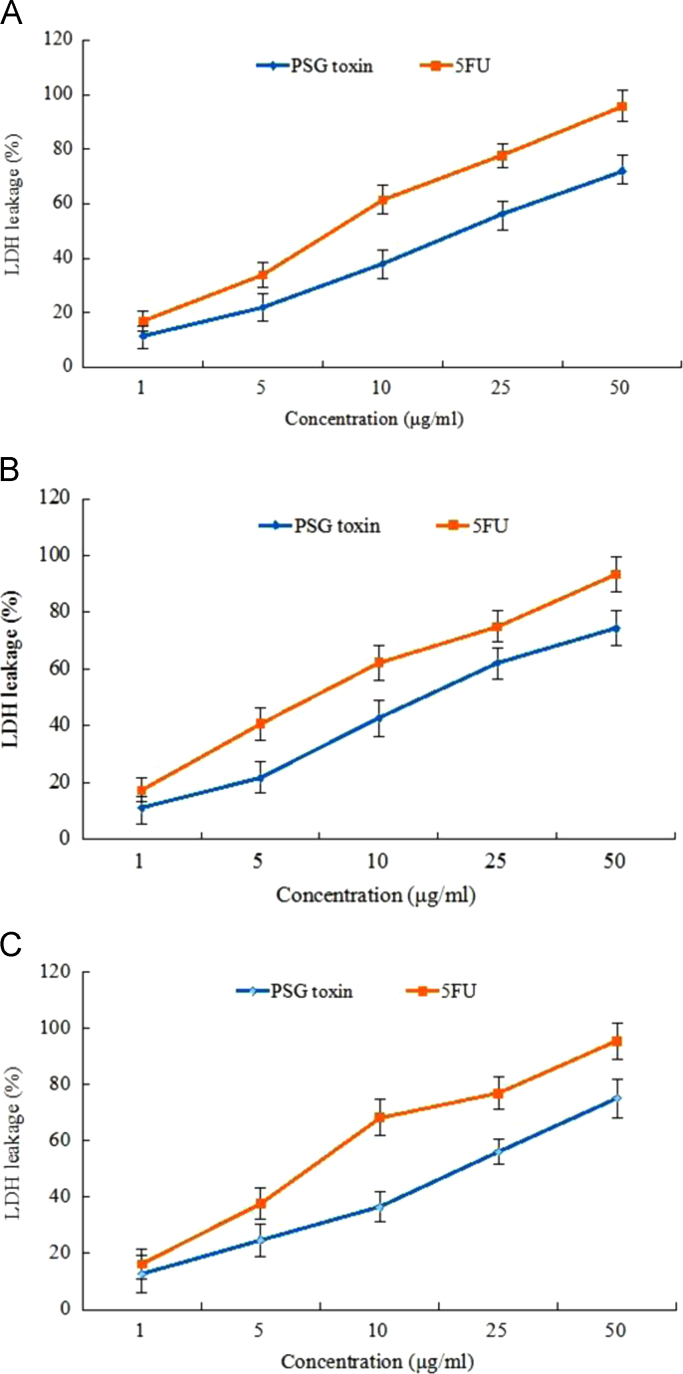
LDH cell viability curve of PSG toxin against (A) KB oral cancer cells (B) HeLa cervical cancer cells (C) A549 lung cancer cells.

The PSG toxin also exhibited commendable LDH leakage against KB oral cancer cells, HeLa cervical cancer cells and A549 lung cancer cells with IC_50_ concentrations of 22.8 μg/ml, 17.45 μg/ml and 23.52 μg/ml respectively (Fig. 3).

## 2 Materials and methods

### 2.1 in vitro cytotoxicity

#### 2.1.1 Lymphocyte migration inhibition assay

The cytotoxicity of the purified PSG toxin against the primary cells peripheral blood mononuclear cells (PBMC) was determined using the lymphocyte migration inhibition assay following the method of Mousseau et al. (2007). The inhibition of leucocyte migration treated with PSG toxin (5,10,50 µg/ml) under the influence of mitogen (lipopolysaccharide) was measured using a microscope ruler [2].

#### 2.1.2 *in vitro* anticancer activity by MTT assay

The cytotoxicity of the purified PSG toxin against selected cancer cell lines was determined by the MTT cell viability assay [3]. The anti-proliferative potentials of the PSG toxin (0.5,1,5,10,25,50 µg/ml) was studied against the adherent cultures of KB (oral), HeLa (cervical) and A549 (lung) cancer cell line and the inhibitory concentrations (IC_50_) were determined.

#### 2.1.3 LDH release assay

The cell viability and membrane permeability of the PSG toxin (0.5,1,5,10,25, 50 µg/ml) against the KB, HeLa and A549 cancer cells was determined using the LDH leakage assay [4]. The inhibitory effect of the PSG toxin on the mitochondrial enzymes, dehydrogenases are evaluated by the levels of LDH released into medium after action on the substrate lactate in the presence of NADH.
