# Supplementary material for: Data supporting the anticancer activity of posterior salivary gland (PSG) toxin from the cuttlefish Sepia pharaonis Ehrenberg (1831)
Source: Data Brief. 2017 May 5;13:295–300. doi: 10.1016/j.dib.2017.05.010 (PMC5470600; doi:10.1016/j.dib.2017.05.010)
Supplement: Supplementary file 1 — Supplementary material [file mmc1.doc]

**Response to reviewers**

Dear Editor,

With reference to your mail on 27^th^ April 2017, we have thoroughly checked the Manuscript No.: DIB-D-17-00294, the format which is compliance with the same format of the journal DIB. So, I request you to kindly proceed towards the publication as early as possible to your esteemed journal.

Thanks and regards,

Saravanan R

**Title of the MS:**

**“Data supporting the anticancer activity of posterior salivary gland (PSG) toxin from the cuttlefish *Sepia pharaonis,* Ehrenberg (1831)**

Ramachandran Karthik^1^, Venkatesan Manigandan^1^, Kumar Ebenezar^2^, Raghavan Vijayashree^3^ and Ramachandran Saravanan^2^*

^1^ Department of Medical Biotechnology, Chettinad Academy of Research and Education,
Kelambakkam-603 103, Tamil Nadu, India

^2^ Faculty of Allied Health Sciences, Chettinad Academy of Research and Education,
Kelambakkam-603 103, Tamil Nadu, India

^3^ Department of Pathology, Chettinad Hospital and Research Institute, Kelambakkam,-603 103, Tamil Nadu, India

***Address of Correspondence**

Dr. Ramachandran Saravanan,

Asst. Professor-Marine Pharmacology,

Faculty of Allied Health Sciences,

Chettinad Academy of Research & Education,

Kelambakkam, Chennai-603 103,

Tamil Nadu, India

E mail: saranprp@gmail.com
